# Targeting endoplasmic reticulum stress signaling in ovarian cancer therapy

**DOI:** 10.20892/j.issn.2095-3941.2023.0232

**Published:** 2023-10-10

**Authors:** Tianqing Yan, Xiaolu Ma, Lin Guo, Renquan Lu

**Affiliations:** 1 Department of Clinical Laboratory, Fudan University Shanghai Cancer Center, Shanghai 200032, China; 2 Department of Oncology, Shanghai Medical College, Fudan University, Shanghai 200032, China

**Keywords:** Endoplasmic reticulum stress, unfolded protein response, ovarian cancer, targeted therapy

## Abstract

The endoplasmic reticulum (ER), an organelle present in various eukaryotic cells, is responsible for intracellular protein synthesis, post-translational modification, and folding and transport, as well as the regulation of lipid and steroid metabolism and Ca^2+^ homeostasis. Hypoxia, nutrient deficiency, and a low pH tumor microenvironment lead to the accumulation of misfolded or unfolded proteins in the ER, thus activating ER stress (ERS) and the unfolded protein response, and resulting in either restoration of cellular homeostasis or cell death. ERS plays a crucial role in cancer oncogenesis, progression, and response to therapies. This article reviews current studies relating ERS to ovarian cancer, the most lethal gynecologic malignancy among women globally, and discusses pharmacological agents and possible targets for therapeutic intervention.

## Introduction

### Ovarian cancer (OC)

OC is the most mortality of gynecologic malignancy worldwide. Epithelial OC (EOC) accounts for approximately 90% of ovarian neoplasm cases^1^. According to GLOBOCAN 2018 database^2^ estimates, 295,400 new cases of OC were diagnosed, and 184,800 deaths due to OC occurred. In China, population aging aggravates the cancer burden in urban and rural areas^3^. Statistics from 2016 indicated an ovarian carcinoma incidence and mortality in China as high as 57,200 cases and 27,200 deaths, respectively^4^. The 5-year overall survival rate is <45% and decreases to 25% for advanced OC^5^. Because of a lack of early screening methods and an absence of clear symptoms during early OC stages, more than 75% of patients are diagnosed in an advanced stage^6^. Debulking surgery with platinum-based chemotherapy is the first-line therapeutic strategy; however, most patients manifest recurrent disease within 18 months and develop drug resistance leading to therapeutic failure^7^. Notably, the histopathology of ovarian tumors is heterogeneous, and each OC subtype bears genetic mutations, which determine the efficacy of molecularly targeted treatments. Currently, targeted therapies such as antiangiogenic drugs (such as bevacizumab, a recombinant humanized monoclonal IgG1 antibody targeting vascular endothelial growth factor-A) or poly(ADP-ribose) polymerase (PARP) inhibitors are clinically applied to improve the outcomes of this malignancy. Nonetheless, this treatment is effective only in patients with homologous recombination deficiencies^8^. Therefore, the identification of molecules responsible for OC development and progression is essential for both early detection and the development of novel therapeutic approaches for OC.

### Endoplasmic reticulum stress (ERS) and the unfolded protein response (UPR)

**Figure 1:**
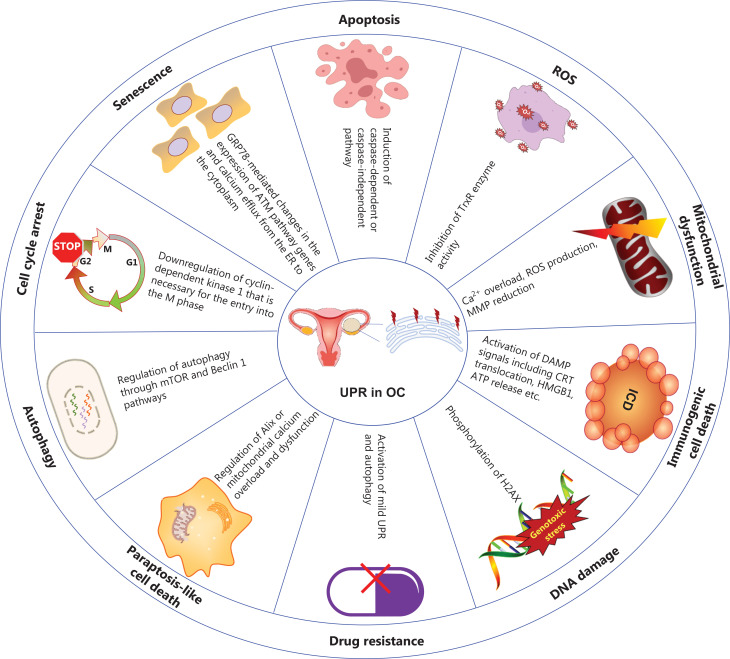
Critical roles of the endoplasmic reticulum unfolded protein response (UPR) in UPR in OC. The UPR is involved in various biological processes in OC that are closely associated with apoptosis^12,13^, ROS^14^, mitochondrial dysfunction^15,16^, non-apoptotic cell death^17,18^, DNA damage^19,20^, drug resistance^21^, autophagy^22^, the cell cycle^23^, and senescence^24^. OC, ovarian cancer; ROS, reactive oxygen species; ICD, immunogenic cell death; MMP, mitochondrial membrane potential; TrxR, thioredoxin reductase; DAMPs, damage associated molecular patterns; CRT, calreticulin; HMGB1, high mobility group protein B1; H2AX, H2A histone family, member X; Alix, apoptosis inducible factor 6 interacting protein; GRP78, glucose regulated protein 78; ATM, ataxia telangiectasia-mutated.

**Figure 2:**
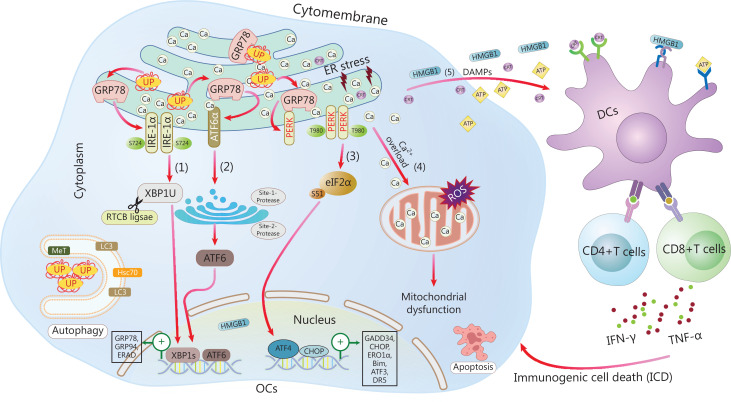
The primary UPR signaling network in OC. In response to the accumulation of unfolded/misfolded proteins (UP), GRP78 dissociates from the 3 UPR sensors (IRE-1, ATF6, and PERK), thus leading to activation of IRE-1, ATF6, and PERK. (1) Activated IRE1α splices XBP1 mRNA into XBP1s. XBP1s translocates to the nucleus and induces the expression of UPR target genes including GRP78 and GRP94, and elicits ERAD or autophagy. (2) Activated ATF6 translocates to the Golgi, where it is cleaved by the site 1 and site 2 proteases, thus generating an active transcription factor. (3) Oligomerized PERK phosphorylates eIF2α and inhibits global translation, but concomitantly induces the expression of ATF4, which in turn activates CHOP expression under extreme conditions, thereby resulting in apoptosis. (4) Intracellular Ca^2+^ translocates from the ER to mitochondria, and ultimately leads to mitochondrial dysfunction and cell apoptosis. (5) DAMP signals, such as CRT translocation, HMGB1, and ATP release, are induced in response to ER stress and activate anti-tumor immunity. ER, endoplasmic reticulum; UP, unfolded or misfolded proteins; IRE1α, inositol-requiring enzyme 1; ATF6, activating transcription factor 6; PERK, protein kinase RNA-like endoplasmic reticulum kinase; XBP1, X-box binding protein-1; GRP78, glucose regulated protein 78; ERAD, ER-associated degradation; DAMPs, damage associated molecular patterns; ICD, immunogenic cell death; eIF2α, eukaryotic translation initiation factor 2α; CHOP, C/EBP-homologous protein; ERO1α, endoplasmic oxidoreductin-1-like protein α; Bim, Bcl-2 interacting mediator of cell death; DR5, death receptor 5; LC3, light chain 3; Hsc70, heat shock protein 70; OCs, ovarian cancer cells; DCs, dendritic cells; TNF-α, tumor necrosis factor α; IFN-γ, interferon γ.

ERS occurs in tumor cells exposed to intrinsic factors (oncogenic activation, chromosome number alterations^9^, and exacerbated secretory capability^10^) and external triggers (hypoxia, nutrient deprivation, and acidosis) that alter protein homeostasis, thus resulting in the accumulation of unfolded or misfolded proteins in the ER lumen. Subsequently, 3 primary UPR signaling pathways, orchestrated by inositol-requiring enzyme 1 (IRE1), activating transcription factor 6 (ATF6), and protein kinase RNA-like endoplasmic reticulum kinase (PERK), are induced, thereby resulting in either adaptive restoration of homeostasis or cell death^11^. The critical roles and signaling networks of the UPR in ovarian carcinoma are illustrated in **Figures 1 and 2**.

#### IRE 1 pathway

IRE1α and IRE1β are 2 isoforms of IRE in mammals. IRE1α is ubiquitously expressed and has been extensively studied, whereas IRE1β is expressed primarily in the gastrointestinal and respiratory tracts^25^. IRE1α is both a kinase and an endo-ribonuclease (RNase), which dimerizes/oligomerizes and auto-trans-phosphorylates under ERS, thus leading to the activation of endo-RNase. Active IRE1α catalyzes the excision of a 26-nucleotide intron within the X-box binding protein-1 (XBP1) mRNA, and RNA-splicing ligase RTCB-mediated ligation of the remaining 5′ and 3′ fragments^26^ shifts the reading frame, thus resulting in translation of a stable and active transcription factor known as XBP1s (spliced form). XBP1s modulates the expression of several UPR target genes involved in ER folding, glycosylation, and ER-associated degradation (ERAD)^27^. In addition, IRE1/RNase activity targets other mRNAs and microRNAs *via* regulated IRE1-dependent decay (RIDD), a novel UPR regulatory pathway that controls cell fate under ERS^28^. In addition to activating ribonuclease activity, IRE1α recruits the adapter target c-Jun N terminal kinase 1 cytoplasmic receptor-associated factor 2 (TRAF2), which in turn activates apoptosis signal-regulating kinase 1 (ASK1) and its downstream target c-Jun N terminal kinase 1 (JNK/MAPK8/SAPK1)^29^. This signaling pathway subsequently activates the nuclear factor-κB (NF-κB) pathway under ERS^30^.

#### ATF6 pathway

ATF6 is a type II transmembrane protein exhibiting transcription factor activity in its cytosolic domain. Under ERS, ATF6 shuttles to the Golgi apparatus and is cleaved by specific site 1 and 2 proteases (S1P and S2P), thus leading to the release of the cytosolic fragment of the protein ATF6. In cooperation with XBP1s, ATF6f up-regulates many genes that increase ER size and protein-folding capability, as well as genes associated with ERAD of misfolded proteins^11,31^. Under irreversible ERS, ATF6 decreases levels of antiapoptotic proteins, such as myeloid cell leukemia-1 (Mcl-1)^32^. Nevertheless, the role of ATF6 in ERS-induced cell death remains to be better explored.

#### PERK pathway

After ERS activation, PERK inhibits global protein translation *via* trans-autophosphorylation and phosphorylation of the eukaryotic translation initiation factor (eIF2α) at serine 51, thereby decreasing the burden of newly synthesized proteins. Furthermore, activating transcription factor 4 (ATF4) mRNA is selectively translated; this mRNA plays an important role in amino acid metabolism, antioxidant response, autophagy, and protein folding^27^. ATF4 expression is also essential for the activation of apoptosis *via* the regulation of C/EBP-homologous protein (CHOP), which upregulates pro-apoptotic members of the B-cell lymphoma-2 (BCL-2) protein family^33^, thereby inhibiting cell growth and promoting DNA damage^19^. Activation of the ATF4-CHOP pathway induces growth arrest and expression of DNA damage-inducible protein 34 (GADD34), an adaptor of eIF2α phosphatase PP1c, which in turn modulates eIF2α dephosphorylation, and recovery from stress or proteotoxicity^34,35^. Nuclear factor erythroid 2-associated factor 2 (Nrf2)^36^ is also phosphorylated by PERK, and consequently transcriptionally up-regulates antioxidants and other components that protect against oxidative stress. The PERK-mediated translational cascade is also required for the activation of NF-κB in cancer cells^37^. Overall, the UPR is a central player in tumor progression^38,39^ representing an attractive therapeutic target in many solid and blood neoplasms^40^. In the next section, we summarize studies associating the UPR with the evolution of ovarian carcinoma.

## Overview of components participating in ERS signaling in OC

Chronic ERS and defective UPR signaling are emerging as critical players in an increasing numbers of human diseases, including OC.

### ER-resident components involved in OC

Multiple molecular chaperones are enriched in the ER, where they ensure normal folding of newly synthesized proteins. The major ER chaperone glucose regulated protein 78 (GRP78) is extensively expressed in human neoplasms. Accordingly, elevated levels of GRP78 in OC tissues are correlated with poor patient prognosis^41^. Functionally, GRP78 is weakly expressed in cisplatin-sensitive OC cells, and it mediates cisplatin-induced senescence^24^. Another ER chaperone protein, disulfide isomerase (PDI), is also highly abundant in OC tissues and predicts poor prognosis in patients diagnosed with OC^41^. Furthermore, tumor suppressor candidate 3 (TUSC3), an ER localized protein responsible for N-glycosylation of proteins, is often lost in epithelial cancers, thus triggering ERS and inducing hallmarks of the epithelial-to-mesenchymal transition (EMT) in OC cells^42^. In our previous study, the UPR signaling component XBP1 was found to be upregulated in OC cell lines. Knockdown of XBP1 significantly inhibits cell propagation and enhances the sensitivity of OC cells to H_2_O_2_ by elevating intracellular ROS levels^43^. Inhibition of the IRE1α/XBP1s branch alone or in combination with immune checkpoint blockade provides a therapeutic strategy for several cancer types with frequent coactivator-associated arginine methyltransferase 1 (CARM1) overexpression, including OC^44^. Furthermore, pharmacological inhibition of the IRE1a/XBP1 pathway alone or coupled with histone deacetylase 6 (HDAC6) inhibition is urgently needed therapeutic strategy against AT rich interactive domain 1A (ARID1A)-mutant OCs^45^. Moreover, key functions of UPR signaling have been established in the regulation of tumor stromal cells. For example, activation of IRE1α-XBP1s reprograms tumor-associated dendritic cells and T cells, thereby impairing anti-tumor activity in OC^46,47^.

### Molecules participating in ERS signaling in OC

Beyond the ER-resident components involved in OC, several molecules have been confirmed to participate in the chemoresistance of OC *via* the ERS signaling. For instance, overexpression of ankyrin repeat domain 1 (ANKRD1) or pleckstrin homology like domain family A member 1 (PHLDA1) in ovarian carcinoma correlates with poor survival, and upregulation of these proteins in OC cell lines modulates cell apoptosis *via* the ERS pathway^48,49^. The ubiquitin-binding protein p62/SQSTM1 (sequestosome 1) is abundant in cisplatin-resistant SKOV3 cell lines and prevents ERS-mediated cell apoptosis, thereby leading to cisplatin resistance. Knockdown of p62 re-sensitizes resistant cells to cisplatin^50^. Twist expression is strongly associated with the expression of DNA damage response proteins, whose upregulation contributes to cisplatin resistance in OC cells. Notably, the combination of niraparib and cisplatin has been found to be considerably effective against 3D cultures of Twist silenced, cisplatin-resistant OC cells with upregulated ERS, thus leading to the initiation of mitochondrially mediated cell death^51^. WW domain-containing oxidoreductase (WWOX), which is frequently lost in several cancers, sensitizes EOC to paclitaxel *via* ERS-induced apoptosis, and is predictive of clinical outcomes in patients^52^. Therefore, ERS response mechanisms can be targeted to resolve chemoresistance in cancer. Additionally, the dysregulation of ubiquitin carboxyl-terminal hydrolase L1 (UCHL1), receptor tyrosine kinase-like orphan receptors (ROR2), and angiotensin II receptor (AGTR1) in OC has been found to predict poor outcomes in patients, thus suggesting that strategies targeting ERS relevant components may provide potential therapeutic benefits^53–55^. All the above factors mediating OC *via* ERS signaling are summarized in **Table 1**. Therefore, targeting UPR components or factors relevant to ERS signaling as a therapeutic strategy to combat ERS-associated pathologies is a promising future research direction.

**Table 1:** Components in ERS signaling implicated in OC

Molecule	Expression	Effects	Reference
ARID1A	Mutated in more than 50% of OCCC	Defining the IRE1a-XBP1 axis of the ERS response as a targetable vulnerability for ARID1A-mutant OCCC	^45^
ANKRD1	Up-regulated in OC cells (*vs.* normal control)	Inducing platinum resistance	^48^
AGTR1	Up-regulated in OC tissues (*vs.* normal tissues)	Correlating with poor outcomes and increasing lipid desaturation *via* SCD1 upregulation, thus ultimately decreasing ERS in multicellular spheroids	^55^
CARM1	Up-regulated in approximately 20% of HGSOC	Hypersensitivity to inhibition of the IRE1α/XBP1s pathway, alone or in combination with immune checkpoint blockade	^44^
GRP78	Up-regulated in OC tissues (*vs.* normal tissues)	Correlating with worse patient survival	^41^
GRP78	Weak in cisplatin-sensitive OC cells	Mediating cisplatin-induced senescence	^24^
PDI	Up-regulated in OC tissues (*vs.* normal tissues)	Correlating with poorer patient survival	^41^
p62	Up-regulated in cisplatin-resistant OC cells (*vs.* cisplatin-sensitive control)	Preventing ERS-induced apoptosis, and leading to cisplatin-resistance	^50^
PHLDA1	Up-regulated in OC tissues (*vs.* normal tissues)	Correlating with poorer patient survival; modulating cell apoptosis *via* the ERS pathway	^49^
ROR2	Down-regulated in HGSOC tissues (*vs.* normal tissues)	Association with HGSOC development and progression; overexpression of ROR2 induces cell apoptosis *via* IRE1α/JNK/CHOP pathway activation	^54^
Twist	Up-regulated in cisplatin-resistant OC cells (*vs.* cisplatin-sensitive control)	Association with cisplatin-resistance and ERS induction, thus leading to initiation of mitochondrial-mediated cell death	^51^
TUSC3	Often lost in epithelial cancers	Association with poor prognosis; loss of TUSC3 alters the molecular response to ERS and induces hallmarks of epithelial-to-mesenchymal transition in OC cells	^42^
UCHL1	Up-regulated in HGSOC tissues (*vs.* normal tissues)	Correlating with poor patient survival; UCHL1 inhibition attenuates mTORC1 activity and induces a terminal ERS response	^53^
WWOX	Frequently lost in several cancers	Mediating the sensitivity of OC cells to paclitaxel *via* modulation of the ERS response	^52^
XBP1	Up-regulated in T cells	Decreasing intra-tumoral T cell infiltration and impairing anti-tumor capability	^47^
XBP1	Up-regulated in dendritic cells	Driving OC progression by blunting anti-tumor immunity	^46^
XBP1	Up-regulated in OC cells	Promoting cell proliferation and decreasing the sensitivity of OC cells to H_2_O_2_	^43^

ANKRD1, ankyrin repeat domain 1; ARID1A, SWI/SNF component; AGTR1, Angiotensin II receptor; CARM1, type I protein arginine methyltransferase; GRP78, 78 kDa glucose-regulated protein; HDAC6, histone deacetylase 6; HGSOC, high grade serous ovarian cancer; OC, ovarian cancer; OCCC, ovarian clear cell carcinomas; PDI, protein disulfide isomerase; PHLDA1, pleckstrin homology-like domain family A member 1; ROR2, receptor tyrosine kinase-like orphan receptors; TUSC3, tumor suppressor candidate 3; UCHL1, ubiquitin carboxyl-terminal hydrolase L1; WWOX, WW domain containing oxidoreductase; XBP1, X-box binding protein-1.

## Studies on pharmacological agents targeting ER homeostasis in OC

UPR signaling is believed to be a self-protection mechanism in cells. Nevertheless, if the intensity or duration of cellular stress is elevated, these pathways instead activate cell death. Therefore, regulation of UPR signaling components has the potential to either stimulate or attenuate protein folding, and to have therapeutic effects in diseases such as diabetes and neurodegenerative diseases, or in the induction of apoptosis, thus enabling anticancer strategies^38^. To date, the mechanisms defining the threshold that switches UPR signals from adaptive cellular protection to proapoptotic cell death or vice versa remain to be elucidated. ERS activation is intricately involved in signaling pathways including cellular autophagy^56,57^, oxidative stress^58,59^, Ca^2+^ homeostasis^60,61^, apoptosis^57^, metabolic disorders^12,62^, and inflammatory responses^30,37^. Thus, clarification of the ERS pathway, and the rationale for drug design and implementation, are key challenges. We next review the pharmacological agents targeting the ERS signaling in ovarian carcinoma.

### ERS-mediated autophagy induced by pharmacological agents resulting in either protective or anti-tumor effects in OC

The UPR is indispensable for the adaptation of cancer cells to rapid growth, hypoxia, nutrition deprivation, and chemotherapies. The UPR restores cellular homeostasis, thereby leading to degradation of unfolded and/or misfolded proteins *via* autophagy or ERAD. Nonetheless, the UPR also results in cell death under certain circumstances^63^. For instance, OC cell apoptosis induced by metformin (a first-line treatment for type 2 diabetes) has been found to be abrogated by autophagy and PERK activation^64^; however, in another study, metformin has been found to promote the apoptosis of OC cells *via* ERS induction^65^. Similarly, quercetin (3,3′,4′,5,7-pentahydroxyflavone) has been reported to induce ERS, thus concomitantly promoting protective autophagy by activating the signal transducer and activator of transcription 3 (p-STAT3)/BCL-2 axis^66^. Intriguingly, one study has demonstrated that quercetin suppresses DNA double-strand break repair and enhances the radiosensitivity of human OC cells *via* a p53-dependent ERS pathway^67^. Another study has indicated that quercetin enhances the apoptosis of OC cells exposed to tumor necrosis factor-associated apoptosis-inducing ligand (TRAIL) by upregulating death receptor 5 (DR5) expression after ERS^68^. Furthermore, the HIV protease inhibitor saquinavir induces ERS-regulated cellular autophagy through the mTOR and Beclin 1 pathway, and decreases the sensitivity of SKOV3 to cisplatin^69^, whereas saquinavir has also been reported to promote cell death in OC cells characterized by ERS activation and autophagy^22^. These contradictory results suggest that a balance may exist between cell death and survival, as mediated by ERS involved in autophagy, according to the degree and duration of drug stimulation. Some pharmacological compounds exert anti-tumor effects *via* induction of ERS and autophagy. For example, the flavonoid kaempferol inhibits cell propagation and induces apoptosis in A2780 cells by triggering ERS-mediated cytotoxic autophagy^56^. B19 (a novel monocarbonyl analogue of curcumin) induces apoptosis in human OC cells *via* activation of ERS^70^ and the autophagy signaling pathway^71^. Trans10, cis12-conjugated linoleic acid (occurring naturally in dairy products and red meat) has also been identified to inhibit the proliferation and migration of OC cells through activating ERS and autophagy^72^. Mifepristone sensitizes OC cells to proteasome or lysosome inhibitors by inducing ERS and autophagic flux^73^. The aforementioned studies have indicated that ERS signaling and autophagy may be used by OC cells to survive in the hostile tumor microenvironment; however, extensive stress and autophagy might result in cell death in OC, thereby suggesting a need for therapeutic strategies targeting ERS signaling or autophagy in cancer therapy.

### Pharmacological agents inducing ERS-mediated anti-tumor effects involve apoptosis and non-apoptotic cell death in OC

As described above, ERS has antipodal functions in the progression of OC. Beyond the protective effects of ERS on OC cell fate, most compounds like ABT-737, GYY4137 and Garcinone E etc. tend to exert anti-tumor effects directly *via* ERS induction^74–82^. ERS mediated OC cell death also includes caspase-dependent^12,15,16,60,83–85^ or caspase-independent cellular apoptosis^13,86–88^, and non-apoptotic cell death such as immunogenic cell death (ICD)^17,89–91^ and paraptosis-like cell death^18,92^.

#### Apoptosis of OC cells mediated by ERS

The 3 main sensors (PERK, IRE1, and ATF6) and their downstream cascades are involved at different levels in cell death induced by unresolved ERS, among which the PERK/ATF4/CHOP pathway plays a critical role in cell destruction^13,20,93–95^. The pharmacological agent cucurbitacin I induces OC cell death *via* CHOP- and caspase-12-dependent ERS-associated apoptosis^86^. α, β-thujone leads to cell death *via* activation of ERS, DNA damage, and caspase-dependent apoptotic pathways^12^. ERS- and caspase-dependent apoptosis is also induced in OC cells treated with pimaric acid^83^ or valosin-containing protein inhibitors^84^. Furthermore, caspase-independent pathways such as the JNK branch of the IRE1 signaling also promote cell death^96^. For example, low levels of glucose and metformin have been reported to induce apoptosis of human OC cells *via* activation of the ERS-associated ASK1-JNK pathway^65^. Sodium 4-carboxymethoxyimino-(4-HPR) (a novel water-soluble derivative of 4-oxo-4-HPR) exhibits anticancer activity against solid tumors *in vivo* and *in vitro* through ERS-activated p-JNK signaling, and fenretinide (a synthetic retinoid) induces apoptosis *via* a ROS-dependent mechanism involving ERS and JNK activation^97–99^.

#### Nonapoptotic cell death mediated by ERS in OC

Beyond ERS-mediated caspase-dependent/independent apoptosis, some agents induce ICD or paraptosis-like cell death. ICD denotes a specific variant of regulated cell death driven by stress and the induction of adaptive immunity against the antigens of dead cells. For instance, ERS induced by thapsigargin or doxorubicin partially regulates the release and binding of calreticulin (CRT, an ER chaperone) to the surfaces of OC cells, where it releases an “eat me” signal and activates anti-tumor adaptive immune responses^89^. CRT exposure on the surfaces of primary and metastatic high grade serous OC cells is driven by a chemotherapy-independent ERS response and culminates in the establishment of a local immune microenvironment characterized by Th1 polarization and cytotoxic activity, thus enabling superior clinical benefits^89^. Benzenesulfonamide (a mitochondrial uncoupler) activates ERS sensors, as well as growth inhibition and apoptosis promotion, thus resulting in ICD and anti-tumor immune effects^17^. Lau et al. have reported that paclitaxel induces ICD-associated damage-associated molecular patterns (DAMPs, such as CRT exposure, ATP secretion, and high mobility group box 1 release) in OC *in vitro* and elicits significant anti-tumor responses in tumor vaccination assays *in vivo*^90^. In addition, paraptosis, first reported in 2000^100^, is a caspase-independent form of programed cell death, characterized by the absence of classical apoptotic features such as apoptotic body and chromatin agglutination^100,101^. The morphological features of paraptosis are also distinct, including swollen ER or mitochondria and cytoplasmic vacuolization^102^. *De novo *synthesis of proteins and ERS are also essential for paraptosis. Morusin (a prenylated flavonoid extracted from the root bark of *Morus australis*) induces paraptosis-like cell death *via* activation of ERS and mitochondrial Ca^2+^ overload and dysfunction in EOC^92^. Another study has found that the novel rhein derivative 4a induces paraptosis-like cell death by ERS in OC cells^18^. Cucurbitacin I has also been proposed to mediate ERS-dependent autophagy, and caspase-independent nonapoptotic cell death^86^. Several pharmacological agents that target ERS signaling for the potential therapy of OC, described above or in prior studies^14,103–109^, are summarized in **Table 2**.

**Table 2:** Pharmacological agents targeting ER homeostasis in OC

Agent	UPR mediator	*In vitro* or *in vivo* model	Effects	Reference
Clinical drugs				
Angiotensin II	GRP78, (p)PERK, CHOP	A2780 and OVCA429 cells, and xenograft models	Promoting MCS formation and peritoneal metastasis of EOC cells, and decreasing ERS	^55^
Apomorphine	GRP78, p-PERK, CHOP	ES2, OV90	Suppressing mitochondrial energy metabolism and inducing ERS	^95^
Bortezomib	ATF3	SKOV-3, OVCAR-3	Mediating ERS, cell cycle arrest, and apoptosis	^23^
Bortezomib	GRP78, PERK, IRE-1, CHOP, Calnexin, Ero1-Lα	Various cancer cells (OC cells including PA1, A2780, A2780/cis, and SKOV3)	Inducing UPR and increasing oncolytic HSV-1 replication, thus resulting in synergistic anti-tumor effects	^110^
Cisplatin	GRP78	A2780, C13K/Cis	Inducing senescence	^24^
Fenretinide	p-JNK	A2780, OVCAR-3	Inducing apoptosis through ROS generation, ERS response, JNK activation, and induction of proapoptotic placental bone morphogenetic protein	^98^
Fenretinide	XBP1, CHOP, GRP78, (p)eIF2α, (p)JNK	A2780, A2780/HPR, IGROV-1, OVCAR-3	Inducing apoptosis of OC cells *via* a ROS-dependent mechanism involving ERS and JNK activation	^99^
Metformin	ATF4, p-PERK, p-eIF2α	PA-1, OVCAR-3	Exerting anticancer effects on OC cells by inhibition of autophagy and PERK	^64^
Metformin	(p)JNK, CHOP, Caspase 4	SKOV3, OVCAR-3, HO8910	Inducing cell apoptosis *via* ASK1-mediated mitochondrial damage and ERS	^65^
Mifepristone	GRP78, CHOP	OV2008, OV2008/cis, SKOV3	Triggering the UPR, increasing autophagic flux, and killing OC cells	^73^
Methiothepin	p-PERK, ATF4, CHOP	ES2, OV90	Exserting anti-cancer effects through regulating expression of ERS-associated proteins and apoptosis	^94^
Nelfinavir	GRP78, PERK, (p)eIF2α, ATF4, IRE-1, CHOP, ATF6	PEO1, PEO4, PEO6, PEO14, PEO23	Inducing UPR, and modulating protein synthesis, DNA damage, lysosomal impairment, and potentiation of toxicity caused by proteasome inhibition	^20^
Niraparib, cisplatin	GRP78, ATF6, CHOP	OV90, SKOV3, OV90/cis, SKOV3/cis	Inducing ERS and apoptosis	^51^
Neratinib	eIF2a	SKOV3, OVCAR3	Killing OC cells through convergent DNA damage and ERS signaling	^88^
Paclitaxel	PERK, (p)eIF2α	ID8 cells and ID8F3 cells (murine model of HGSOC)	Inducing immunogenic cell death and ERS	^90^
Saquinavir	GRP78	SKOV3	Inducing ERS, decreasing the sensitivity of DDP in SKOV3	^69^
Saquinavir	GRP78/ATF6	A2780, SKOV3, CAOV3, OVCAR3, etc.	Inducing ERS, autophagy, and apoptosis	^22^
Other herbal extracts or synthetics				
Alpinumisoflavone	GRP78/p-eIF2α/IP3R1/VDAC	ES2, OV90	Inhibiting cell proliferation and migration, and promoting apoptosis	^111^
AB23	GRP78, IRE-1, p- eIF2α	A2780, A2780/taxol, HEY	Inducing apoptosis and ERS	^112^
Arginase-1	PERK, ATF4, CHOP, p-eIF2α	Various cancer cells (OC cells including OVCAR-3)	Inducing ERS-mediated cell apoptosis	^113^
ABT737	PDI, GRP78, CHOP	SKOV3/cis, COC1/cis, A2780/cis	Reversing cisplatin resistance by regulating ER-mitochondrial Ca^2+^ signal transduction in human OC cells	^74^
α, β-thujone	GRP78, p-PERK, ATF4, CHOP	ES2, OV90 cells	Regulating multiple intracellular stress-associated metabolic reprogramming and caspase-dependent apoptotic pathways	^12^
Benzenesulfonamide	ATF3/6, CHOP, GADD34, PERK	A2780, patient-derived EOC cell lines, and a mouse model	Activating ERS and anti-tumor adaptive immune responses, inducing apoptosis and immunogenic cell death	^17^
B19	GRP78, XBP1, ATF4, CHOP	A2780, CP70	Inducing apoptosis in OC cells *via* ERS and ROS production	^70^
B19	PDI, GRP78, CHOP, ATF6, XBP1	HO8910	Inducing human OC cell apoptosis *via* activation of ERS and the autophagy signaling pathway	^71^
BH3 mimetic S1	(p)JNK, GRP78, PDI, Caspase-4	SKOV3, SKOV3cis	Inducing ERS-associated apoptosis in cisplatin-resistant human OC cells	^78^
BHPI	GRP78, p-eIF 2α, CHOP, IRE-1, PERK, XBP1, ATF6	Various cancer cells (OC cells including OVCAR-3, IGROV-1, and ES2)	Activation of the UPR, inducing tumor regression	^82^
Campesterol	p-PERK, p-eIF2α, p-IRE1α, CHOP, ATF6, GRP78	ES2, OV90 cells	Suppressing cell proliferation, cell cycle progression, and cell aggregation, inducing cell apoptosis	^114^
Copper (II)-phenanthroline complexes	GRP78, PERK, IRE-1, CHOP	A2780	Inducing ERS and subsequently cell death mediated by UPR	^79^
CD437	eIF2α, ATF4, XBP1, BIP, GADD34 and CHOP	KF, SHIN-3, KOC-2S, SKOV3, TU-OS-3	Inducing apoptosis through specific ERS pathways	^80^
Cucurbitacin-I	GRP78, (p)PERK, IRE-1, (p)eIF2α, ATF6, CHOP	SKOV3	Inducing cancer cell death through the ERS pathway	^86^
Cadmium	GRP78, (p)PERK, (p)eIF2α	COV434	Inducing ovarian granulosa cell damage by activating ERS	^103^
Coenzyme Q0 (isolated from *Antrodia camphorate*)	Caspase-12, HSP-70	SKOV3, A2780, CP70 and IOSE cells, and a xenografted tumor model	Inducing apoptosis through mitochondrial and ERS pathways	^104^
DPP23	GRP78/IRE1α/XBP1/ATF4/CHOP	A2780, A2780/cis	Overcoming multi-resistance by inducing ERS in cisplatin-resistant A2780/Cis OC cells	^21^
DWP05195	CHOP	A2780	Inducing ERS-dependent apoptosis through the ROS-p38-CHOP pathway in human OC cells	^77^
Epoxycytochalasin H	GRP78, Caspase 4	A2780	Inducing apoptosis in A2780 cells through mitochondrial damage and ERS	^85^
ERX-41	p-PERK, PERK, p-eIF2α, eIF2α, CHOP, XBP1	OC and other cancer cell lines	Inducing ERS and subsequently cell death	^115^
FCCP	p-eIF2α, ATF4/5, CHOP	A2780 and ID8 cells, and a mouse tumor model	Cooperating with ERS to facilitate the response to chemotherapeutics in OC	^116^
Fucoidan	GRP78, IRE1α, ATF6, PERK, CHOP, p-eIF2α	ES2 and OV90 cells, and a zebrafish xenograft model	Perturbing Ca^2+^ homeostasis, inducing ERS and OC cell death	^57^
Fucosterol	GRP78, IRE1α, ATF6, (p)PERK, CHOP, eIF2α, (p)eIF2α, (p)JNK	ES2 and OV90 cells, and xenograft models	Inducing mitochondrial dysfunction and ERS	^15^
Garcinone E	IRE-1, XBP1	HEY, A2780, and A2780/taxol	Triggering ERS, inducing apoptosis, and inhibiting migration and invasion in OC cells	^76^
Glutaminase inhibitor compound 968	PERK, Calnexin, GRP78	IGROV-1, SKOV3, HEY	Inhibiting cell growth in OC cells through induction of G1 phase cell cycle arrest, apoptosis, and cellular stress	^58^
GYY4137	IP3R, ATF4, CHOP, XBP1, NRF2F2	A2780	Inducing ERS and apoptosis	^75^
Gold(I)-phosphane dithiocarbamate complexes	(p)PERK, p-eIF2α, GRP78, CHOP	A2780, A2780cis	Triggering an ERS-dependent immune response in OC cells	^91^
Gold(I) complex containing an oleanolic acid derivative (4b)	(p)PERK, Calnexin, GRP78 ATF4, CHOP	A2780	Inducing A2780 cell apoptosis by activating ERS	^14^
Hesperidin	GRP78, CHOP	A2780	Inhibiting cell viability and apoptosis *via* ERS signaling pathways	^117^
Indole-3-carbinols (I3C)	ATF3, CHOP	OVCAR3, OVCAR5, OVCAR8, A2780, SKOV3, 3A, HEY, and CAOV3 cells, and a xenograft mouse model	Inducing profound cell cycle arrest, apoptosis, and disruption of multiple pathways including those regulating ERS, the cytoskeleton, chemoresistance and carcinogen metabolism, after combination treatment with bortezomib and I3C	^62^
Isoliquiritigenin	p-eIF2α, CHOP, GRP78, XBP1	SKOV3	Inducing SKOV-3 cell apoptosis	^105^
JI017	GRP78, (p)PERK, (p)eIF2α, ATF4, CHOP	A2780, OVCAR-3	Inducing apoptosis *via* the Nox4-PERK-CHOP axis in OC cells	^13^
Kaempferol	GRP78, PERK, ATF6, IRE-1	A2780	Inhibiting cell proliferation and inducing apoptosis in A2780 cells by triggering ERS-mediated cytotoxic autophagy	^56^
Laminarin	IRE1α, p-PERK, p-eIF2α, GRP78, CHOP, ATF6, XBP1	ES2 and OV90 cells, and a xenograft model	Suppressing the growth of OC cells *via* mitochondrial dysfunction and ERS	^16^
Myricetin	GRP78, CHOP	SKOV3	Inducing DSBs and ERS, thus leading to apoptosis in SKOV3 cells	^87^
Morusin	GRP78, CHOP, IRE-1, p-eIF2α	A2780, SKOV3, and HO8910 cells, and tumor xenograft	Inducing paraptosis-like cell death *via* mitochondrial Ca^2+^ overload and dysfunction in EOC	^92^
MDA-7/IL-24	p-PERK, p- eIF2α	SKOV3, OVCAR	Inducing ERS and activating multiple proapoptotic pathways	^106^
Novel rhein derivative 4a	GRP78, PERK, eIF2α, ATF4	A2780, SKOV3	Inducing paraptosis-like cell death by ERS	^18^
O6-Benzylguanine	Caspase-12	SKOV3, SKOV-3x	Enhancing cisplatin cytotoxicity and apoptosis *via* the ERS pathway	^118^
PABA/NO	PDI, GRP78, p-eIF2α, GADD34, HSP-70, CHOP, XBP1	SKOV3	Activation of the UPR, inducing anti-tumor activity	^81^
Pimaric acid	(p)PERK, IRE-1, ATF4, CHOP	T1074, PA-1	Exerting anti-cancer effects *via* ERS, caspase-dependent apoptosis, cell cycle arrest, and inhibition of cell migration	^83^
PPARγ agonist	GRP78, CHOP	SKOV3	Inducing ERS-mediated apoptosis	^107^
Quercetin	GRP78, CHOP, Caspase-4	CAOV3 cells and mouse xenograft model	Inducing ERS, apoptosis and protective autophagy	^66^
Quercetin	p-eIF2α, CHOP	OV2008, A2780, GM9607 cells, and xenograft model	Suppressing DNA double-strand break repair and enhancing the radiosensitivity of human OC cells *via* a p53-dependent ERS pathway	^67^
Quercetin	p-JNK, JNK, GRP78, and CHOP, p-eIF2α	SKOV-3, OVCAR-3, TOV21G, HOSE cells, and xenograft model	Enhancing apoptotic death of OC cells due to TRAIL through upregulation of CHOP-induced DR5 expression after ROS mediated ERS	^68^
RA375	CHOP, XBP1	ES2, SKOV3 cells, and xenograft model	Producing ERS and oxidative stress, and triggering apoptosis	^59^
RGD-CaPO/DOX NPs	GRP78, p-eIF 2α, CHOP	SKOV3 and mouse model	Aggravating ERS, Ca^2+^ overload, and mitochondrial dysfunction, thus ultimately triggering mitochondrial apoptosis	^60^
RA183	CHOP, GRP78, XBP1, ATF4	OV2008, OVCAR-3 cells, and mouse model	Triggering unresolved ERS and apoptosis	^108^
STF-083010	XBP1, p-PERK, ATF-4, CHOP	OVCAR3, SKOV3	Inducing ERS-mediated cell apoptosis	^119^
Sodium 4-carboxymethoxyimino-(4-HPR)	p-JNK, p-eIF2α	A2780, IGROV-1, SKOV3, and mouse xenograft models	Inducing ERS-mediated cell death	^97^
Tunicamycin	CHOP	SKOV3	Inducing ERS-regulated proliferation, migration, and invasion of SKOV3 cells	^120^
TAT-IDPS	Calpain-1, PDI, CHOP, Caspase-4	SKOV3/cis	Inducing ERS-mediated apoptosis	^121^
Titanocene difluorides	CHOP, GRP78, Caspase-12	A2780, A2780/cis, SKOV3	Perturbing ER homeostasis, activating autophagy, and triggering an alternative cell death pathway	^63^
Trans10, cis12 conjugated linoleic acid	ATF4, CHOP, GADD34	A2780, SKOV3	Inhibiting proliferation and migration of OC cells by inducing ERS, autophagy, and modulation of Src	^72^
Thapsigargin/doxorubicin	Calreticulin (CRT)	OVCAR3, SKOV3, A2780	Partly regulating the release and binding of CRT to cancer cells, in which CRT may play a role in immunogenic cell death	^89^
TRIP complexes	CHOP	Mice bearing A2780 OC xenografts	Triggering ERS-induced cancer cell death	^109^
2-deoxy-D-glucose	GRP78, CHOP	SKOV3	Sensitizing SKOV3 cells to cisplatin by increasing ERS and decreasing ATP stores in acidic vesicles	^122^
4-Methylumbelliferone	ATF6, GRP78, CHOP	ES2, OV90	Disrupting Ca^2+^ homeostasis and inducing ERS	^61^

AB23, alisol B 23-acetate; B19, novel monocarbonyl analogue of curcumin; BHPI, 3,3-bis(4-hydroxyphenyl)-7-methyl1,3-dihydro-2H-indol-2-one; CD437, synthetic retinoid; Cis, cisplatin; DPP23, synthetic chalcone derivative (E)-3-(3,5-dimethoxyphenyl)-1-(2-methoxyphenyl)prop-2-en-1-one; DWP05195, transient receptor potential vanilloid 1 antagonist; DSB, DNA double-strand break; FCCP, mitochondrial uncoupler carbonyl cyanide p-trifluoromethoxyphenylhydrazone; HGSOC, high grade serous ovarian cancer; JI017, complex herbal medication; OC cells, ovarian cancer cells; PPARγ, peroxisome proliferator-activated receptors γ; PABA/NO, O2-[2,4-dinitro-5-(N-methyl-N-4-carboxyphenylamino) phenyl]1-(N,N-dimethylamino) diazen1-ium-1,2-diolate; ROS, reactive oxygen species; RGD-CaPO/DOX NPs, Arg-Gly Asp (RGD)-modified amorphous Ca^2+^ phosphate loaded with doxorubicin; RA183, compound generated from substitution of the m, p-chloro groups of bis-benzylidinepiperidone RA190 for p-nitro; STF-083010, novel inhibitor of IRE1α; TAT-IDPS, TAT-fused inositol 1,4,5-trisphosphate receptor-derived peptide; TRIP, tricarbonyl rhenium isonitrile polypyridyl; UPS, ubiquitin-proteasome system; VCP, valosin-containing protein).

## Concluding remarks and future perspectives

On the basis of *in vitro* and *in vivo* experiments, the activation of UPR has been shown to modulate processes including the cell cycle, oxidative stress, autophagy, cell death, and chemoresistance in OC (**Figure 1**). This review summarizes studies on UPR components and pharmacological compounds that target ERS-associated pathways in OC. Small molecules that specifically target components of the UPR signaling network are promising potential therapeutic interventions. Therefore, the UPR is emerging as an appealing therapeutic target; however, the benefits and risks of modulating the UPR in any tumor type require further evidence. Numerous compounds are being developed to target the 3 UPR sensors; however, the factors determining the behavior of a particular sensor as a pro- or anti-apoptotic signal remain unclear. On the one hand, cancer cells use adaptive responses to survive excessive stress, which are accompanied by tumor initiation, progression, metastasis, immune escape, and chemoradiotherapy resistance. On the other hand, excessive or sustained stress results in tumor killing^123^. Strategies for blocking tumor stress relief or elevating stress-induced cell mutation may achieve optimal therapeutic outcomes. The new compound ERX-41 has been documented to exacerbate ERS, thus leading to several types of cancer deaths with elevated ERS^115^. The concept of increasing ERS by ERX-41 in cancer cells for therapeutic purpose has been licensed to Dallas-based EtiraRx, and is expected to enter clinical trials soon. Moreover, monitoring the adaptive response on multiple scales is necessary to help design optimal treatment schedules and balance on-target toxicity with tumor eradication. Finally, potential combinatorial therapies with clinical chemotherapeutic drugs are also appealing and promising. Future studies addressing these issues are expected to pave the way to novel avenues for treating ERS-associated diseases.
